# Multicultural doula support and obstetric and neonatal outcomes: a multi-centre comparative study in Norway

**DOI:** 10.1186/s12884-024-07073-y

**Published:** 2024-12-24

**Authors:** Hanna Oommen, Linda Reme Sagedal, Jennifer J. Infanti, Ulrika Byrskog, Marit Stene Severinsen, Mirjam Lukasse

**Affiliations:** 1 Department of Research, Sørlandet Hospital Kristiansand, Kristiansand, Norway; 2 Department of Obstetrics and Gynecology, Sørlandet Hospital Kristiansand, Servicebox 416, 4604 Kristiansand, Norway; 3 The Research Center for Women’s, Family and Child Health, Faculty of Health and Social Sciences, University of South-Eastern Norway, Kongsberg, Buskerud Norway; 4 Institute of Clinical Medicine, University of Oslo, Oslo, Norway; 5 Department of Public Health and Nursing, Faculty of Medicine and Health Sciences, Norwegian University of Science and Technology, Trondheim, Norway; 6 School of Health and Welfare, Dalarna University, Dalarna, Sweden; 7 Church City Mission, Oslo, Norway

**Keywords:** Health Promotion, Migrants, Health Disparities, Perinatal Care, Childbirth

## Abstract

**Background:**

Migrant women face an increased risk of poor obstetric and neonatal outcomes. Norway implemented a multicultural doula (MCD) program in 2018, which was designed to improve pregnancy care for this group in vulnerable circumstances. This study aimed to assess the impact of MCD support, provided in addition to standard care, on obstetric and neonatal outcomes for selected newly arrived migrants.

**Methods:**

This was a multi-centre case–control study involving all nine hospitals actively running the MCD program, which covers four of Norway’s five regions. Women who received MCD support at the time of childbirth (*n* = 339), from 2018–2023, were compared to similar newly arrived immigrant women who did not receive MCD support (*n* = 339) and gave birth within the same timeframe. Hospital records were reviewed, and outcomes were analysed using binary logistic regression. The results are expressed as crude and adjusted associations with 95% confidence intervals (CIs).

**Results:**

Women receiving MCD support exhibited a 41% lower likelihood of undergoing emergency caesarean sections (adjusted odds ratio [aOR] 0.59, 95% Cl 0.34–0.98) and those giving birth vaginally had a 75% lower risk of estimated blood loss ≥1000 ml (aOR 0.25, 95% Cl 0.12–0.52) compared with women without MCD support. Additionally, MCD support was associated with more use of pain-relief (aOR 2.88, 95% Cl 1.93–4.30) in labour and increased rates of exclusive breastfeeding at discharge (aOR 2.26, 95% Cl 1.53–3.36).

**Conclusions:**

Our study suggests that MCD support may contribute to improved outcomes for migrants in vulnerable circumstances, potentially impacting their future reproductive health and children’s well-being.

**Supplementary Information:**

The online version contains supplementary material available at 10.1186/s12884-024-07073-y.

## Background

The global increase in childbearing migrant women [1, 2] highlights the need to understand their reproductive health and provide targeted interventions to address health inequalities. Norway has seen significant migration growth in recent decades. By early 2023, 16% of resident women (428,100) were migrants, predominantly from Europe, Asia, and Africa [3]. Over one-quarter had lived in Norway for five years or less, while approximately 8.5% were resettlement refugees, typically with lower educational levels compared to other migrants and the general population [3].

Newly arrived migrant women are often considered vulnerable due to their heightened risk of adverse health outcomes, compounded by challenges such as socioeconomic instability, limited healthcare access, and cultural or linguistic barriers. In this study, vulnerability refers to the increased susceptibility of migrant women to poor perinatal outcomes. Specifically, a pregnant woman in vulnerable circumstances is defined as one who faces physical, psychological, cognitive, and/or social risk factors combined with a lack of adequate support and/or coping skills [4]. This vulnerability places migrant women at increased risk of various adverse perinatal outcomes including hyperemesis [5], preterm births [6, 7], stillbirths [7], caesarean sections [8, 9], placental abruption [10], severe maternal morbidities (near-miss) [11], obstetric anal sphincter injuries [12], postpartum haemorrhage (PPH) [13], and postpartum depression [5]. Furthermore, the use of epidural analgesia during labour is less common among migrants [14].

Length of stay since migration and the Human Development Index in migrants’ countries of origin are linked to perinatal outcomes [7, 12, 13, 15]. The “Healthy Immigrant Effect” suggests that migrants, including asylum-seeking women, may initially exhibit better health outcomes than native populations, because of the healthy selection of those able to migrate [16, 17]. However, this advantage often diminishes due to various pre- and post-migration factors, such as limited access to healthcare, poor living conditions, and high levels of stress [15, 16, 18–21]. Newly arrived asylum-seekers and undocumented migrants are particularly in vulnerable circumstances as they face the complexities of asylum processes, limited social networks, employment opportunities, suboptimal living conditions in host countries, acculturation processes, language barriers, and discrimination [18, 19, 22]. Migrants originating from conflict zones may also experience additional burdens, including fear of persecution and exposure to trauma [18, 19].

Cultural support involves providing care, guidance, and resources that are sensitive to the cultural, linguistic, and social backgrounds of the individuals being supported [23, 24]. In the context of this study, this includes understanding and respecting the cultural practices, beliefs, values, and languages of migrant women, and incorporating these elements into their maternity care to ensure they feel understood, respected, and empowered. The importance of cultural support in childbirth is frequently underestimated, particularly in healthcare systems that prioritise biomedical approaches and standardised maternity care, often overlooking the cultural and emotional needs of the mother [25, 26].

Multicultural doulas (MCDs) are trained laywomen from migrant communities who provide culturally aware physical, emotional, and informational support [22]. They help bridge gaps in understanding, advocate for the mother’s voice, and ensure her active involvement, while also assisting with navigating unfamiliar maternity care systems during labour and the perinatal period [19–21]. Their support comes in addition to standard care, which in Norway includes the use of professional interpreters over the phone [27]. MCDs have been shown to assuage women’s concerns, increase awareness of danger signs, lower the threshold for seeking help, enhance cultural sensitivity during childbirth, and foster continuity of care and trust in healthcare systems and providers [28–30]. When women feel that their cultural identity is acknowledged and valued, they are more likely to engage positively with the support offered [31]. Studies conducted in the USA have shown that doula-assisted migrant mothers have lower rates of caesarean deliveries, fewer low-birth-weight babies, reduced birth complications, and higher breastfeeding rates [32–34]. In contrast, a Swedish register study revealed none of these outcomes, demonstrating instead reduced pharmacological pain-relief usage in nulliparous migrant women, increased induction rates, and longer post-birth hospital stays [35]. These contrasting findings highlight the complexity of evaluating doula support across different healthcare contexts, making it compelling to investigate the influence of Norway’s unique MCD program.

Norway’s MCD program, established in 2018, included 120 doulas speaking 23 languages by the close of 2023, serving in nine hospitals. While no quantitative studies on this program have been published yet, a qualitative study revealed that women in the program felt safeguarded and understood [28]. Another study found that doulas themselves view their role as crucial in bridging cultural gaps between migrant women and Norwegian maternity services [36]. The aim of the current study is to assess the influence of MCD support, alongside standard care, on obstetric and neonatal outcomes among selected newly arrived migrants in Norway.

## Material and methods

### Study design

We performed a retrospective multi-centre comparative study using a case–control sampling method to select participants. This study compares obstetric and neonatal outcomes of two newly arrived migrant groups: those who received MCD support during routine pregnancy care and childbirth and those without MCD support.

### MCD support

The MCD program was initiated in Norway 2018 to provide migrant women in vulnerable circumstances with dignified childbirth care and to address the challenges these women face during childbirth [36, 37]. The program trains MCDs through a 56-h course (supplementary file 1) to provide physical, emotional, and informational support to women during pregnancy, childbirth, and the immediate postpartum period [36, 37]. The MCD’s Norwegian language skills are tested in an interview and must be sufficient for participation in the course. The criteria for women to receive support from an MCD through the program are: (1) ≤ 5 years of residence in Norway, (2) a limited social network, and (3) poor Norwegian language skills. In selected cases, migrant women receive MCD support when meeting only two of the three criteria, for example if they are single parents or considered in extra vulnerable circumstances due to pre-migration trauma. Eligible expectant mothers are paired at approximately pregnancy week 30 with a primary MCD and a reserve MCD, both of whom speak the same language as the mother-to-be, come from the same geographical area, and are familiar with her cultural background, rituals, traditions, religious, and social needs [36]. In addition to training, MCDs must have experienced childbirth in Norway to ensure familiarity with the Norwegian health system [36]. The MCDs are allocated 24 h (increased to 30 h starting in 2023) of paid time per mother-to-be to provide support. Ideally, half of this time is utilised from the onset of the active phase of labour until the transfer to the maternity ward. However, if the MCD's working day lasted more than 12 h, a reserve doula was called to continue providing support. On average, MCDs provided approximately ten hours of support per woman during pregnancy and the postpartum period, and an additional ten hours during childbirth.

### Data collection

Data were collected from all women (*n* = 339) included in the MCD program between January 1, 2018, and December 31, 2023. Women were defined as having received MCD support when they were allocated an MCD during pregnancy and had an MCD present during childbirth. Women without an MCD present during childbirth (*n* = 3) were excluded from the study. A flowchart showing the reasons for exclusion from the study (*n* = 8) can be found in Supplementary File 2.

For each woman who received MCD support, a control woman was selected from the same hospital’s electronic records, based on the closest possible time of childbirth. Further matching was done based on how long they had been in Norway and their language skills. Migrants in Norway are given a temporary ID before they receive a permanent Norwegian ID, which helped identify recent arrivals. The need for an interpreter was used to determine the woman’s language difficulties.

### Setting and study population

Nine hospitals participate in the MCD program and are included in the present study: Oslo University Hospital (Ullevål Hospital and Rikshospitalet), Akershus University Hospital, St. Olavs Hospital, Haukeland University Hospital, Stavanger University Hospital, Sorlandet Hospital and Vestre-Viken Hospital (Drammen Hospital and Baerum Hospital). These hospitals provide care for women in four of Norway's five regions, and include women from both urban and rural settings [38]. In 2023, 51% of newborns in Norway were delivered at the participating hospitals [38]. It is important to note that the availability of Ukrainian-speaking MCDs varied across hospitals during the study period, which is significant given the influx of Ukrainian refugees to Norway during this time.

### Study measures

The following data were extracted from hospital records: patient demographics (age, marital status, education, country of origin, time since immigration, employment, need for an interpreter, reason for migration, partner’s country of origin, parity, pre-pregnancy body mass index (BMI), female genital mutilation (FGM/C), delivery site in Norway, support person during labour (other than doula), labour characteristics, labour analgesia details (non-pharmacological and pharmacological), delivery outcomes including estimated bleeding, Apgar score, newborn weight and breastfeeding details. PPH was defined as estimated blood loss of ≥ 1000ml.

### Data *analysis*

Descriptive analysis included frequencies and proportions. Obstetric outcomes were compared between groups using Pearson’s chi-square test. The associations between groups and diverse maternal and neonatal outcomes were examined by computing crude and adjusted odds ratios (ORs) with 95% confidence intervals (CIs) via binary logistic regression analysis. The following covariates were used in all multivariable analyses: parity, age, education, prepregnancy BMI, country of origin, and delivery site in Norway. The countries of origin were categorised into three groups: 1) Sub-Saharan Africa, 2) Middle East, Central Asia and South Asia, and 3) Eastern Europe. In some analyses we additionally adjusted for induction of labour, and oxytocin use. Oxytocin use and amniotomy can be part of an induction process or procedures used in prolonged spontaneous labour [39]. Additionally, oxytocin use may potentially affect pain relief usage, mode of delivery, and estimated blood loss [39]. We adjusted for mode of delivery as it is a known factor to influence Apgar Score and breastfeeding practice [40]. The level of significance was set at *p* < 0.05. The data were analysed using SPSS version 25 for PC (IBM Corporation, Armonk, NY, USA) and R version 3.6 (R Core Team, 2020).

## Results

A total of 678 pregnant women were included in the study with equal numbers in both groups: 339 in the group with MCD support (cases) and 339 in the group without MCD support (controls). These participants represent a diverse cohort, originating from 52 countries and communicating in 36 languages. The detailed language distribution is available in Supplementary File 3. Table 1 compares the characteristics of women with and without MCD support. Those receiving MCD support exhibited a higher prevalence of limited education (*p* = 0.005) and unemployment (*p* = 0.012). A minority of women (13%) were from Eastern Europe, with those without MCD support displaying a greater likelihood of originating from this region (*p* = 0.005).

**Table 1:** Characteristics among women with and without Multicultural Doula (MCD) support, *N* = 678

	**MCD support (*n* = 339)**	**No MCD support (*n* = 339)**	***P* value**
Age, *n* (%)			
< 20 years	7 (2)	3 (1)	0.167
20–30 years	171 (50)	149 (44)	
31–40 years	145 (43)	169 (50)	
> 41 years	16 (5)	18 (5)	
Marital status, n (%)			
Married	274 (81)	257 (76)	0.134
Unmarried partner	29 (9)	45 (13)	
Single	36 (11)	37 (11)	
Education, *n* (%)			
No formal education	104 (31)	71 (21)	0.005
Secondary/primary school	115 (34)	106 (31)	
High school	56 (17)	72 (21)	
Higher education	64 (19)	90 (27)	
Country of origin, n (%)^a^			
Sub-Saharan Africa	128 (38)	137 (40)	0.005
Middle East, Central & South Asia	178 (53)	144 (43)	
Eastern Europe	33 (10)	58 (17)	
Female Genital Mutilation (FGM/C), n (%)	40 (12)	53 (17)	0.128
Missing	15 (4)	19 (6)	
Time since immigration, *n* (%)			
≤ 5 years	326 (96)	314 (93)	0.045
> 5 years	13 (4)	25 (7)	
Employment, *n* (%)			
Unemployed	310 (91)	289 (85)	0.012
Employed	29 (9)	50 (15)	
Need for an interpreter,* n* (%)	322 (95)	326 (96)	0.455
No need for an interpreter	17 (5)	13 (4)	
Reason for migration, *n* (%)			
Asylum seeker or refugee^b^	278 (82)	225 (66)	< 0.001
Family reunification	39 (12)	81 (24)	
Education/work	21 (6)	33 (10)	
Missing	1	NA	
Partner’s country of origin			
Same as partner	305 (95)	311 (95)	0.820
Different from partner	16 (5)	15 (5)	
Missing	18 (5)	13 (4)	
Parity, n (%)			
Primiparous	133 (39)	129 (38)	0.903
Multiparous (para 1)	119 (35)	118 (35)	
Multiparous (para ≥ 2)	87 (26)	92 (27)	
Delivery site in Norway			
Southern Region	85 (25)	88 (26)	0.976
Western Region	73 (22)	71 (21)	
Central Region	19 (6)	21 (6)	
Eastern Region	162 (48)	159 (47)	
Pre-pregnancy BMI (mean, SD)	24.73 (4.99)	25.09 (4.94)	0.441
Support person during childbirth other than doula, n(%)			
Partner/relative/friend	189 (56)	227 (68)	< 0.001
None	151 (44)	104 (31)	
Missing	1 (0.3)	6 (2)	

^a^Stateless mothers (*n* = 3) have been categorised based on language. Five mothers (two with MCD and three without MCD support) coming from Middle- or South America are included in the Eastern European group

^b^Includes undocumented migrants *n* = 3 (2 received MCD support)

The primary reasons for immigration in both groups were seeking asylum and refugee status, which constituted 74% of the participants. A significant majority (94%) of the participants had immigrated to Norway within the past five years. A small percentage (4%) of mothers received MCD support even though they had > 5 years since immigration to Norway. Similarly, 25 mothers (7%) with > 5 years since immigration were included in the comparison group. Both groups included more multiparous women (61%) than primiparous women. Almost one-third (31%) of women without MCD support did not have any support person present during childbirth aside from healthcare staff. Other birth companions present at birth in both groups are presented in Table 1. The average duration of MCD support during the active phase of childbirth was 7 h and 36 min (SD 4.11).

After adjusting for parity, age, education, prepregnancy BMI, maternal region of birth, and delivery site in Norway, women with MCD support exhibited a trend toward a lower occurrence of preterm births (< 37 weeks of gestation) (*p* = 0.05) and a significantly higher frequency of inductions (*p* = 0.005) (Table 2). Furthermore, women without MCD support presented with maternal-foetal considerations as the primary reason for admission significantly more frequently than those with MCD support (*p* = < 0.001) (Table 2). After additionally adjusting for induction of labour and oxytocin use, we found that MCD-supported women also received both non-pharmacological (*p* = < 0.001) and pharmacological pain-relief methods (*p* = 0.04) to a greater extent than those without MCD support (Table 2). There was no significant difference in the use of epidural between the groups. A detailed list of pain-relief methods used during childbirth in both groups is presented in Table 3. Migrants receiving MCD support were significantly more active and frequently changed their positions during labour (*p* = < 0.001). They also received massage (*p* = < 0.001) and opiates (*p* = < 0.001) more often than mothers who did not receive MCD support.

**Table 2:** Associations of obstetric factors during the first stage of labour, comparing women with and without Multicultural Doula (MCD) support, *N* = 678

	**MCD** **Support (*n* = 339)**	**No MCD support (*n* = 339)**	**Univariable analysis OR or MD (95% CI)**	**Multivariable analysis Adjusted OR (95% CI)**
Primary reason for admission, *n* (%)				
Early labour	58 (17)	62 (18)	0.98 (0.66–1.46)	0.97 (0.63–1.51)^a^
Active labour*	138 (40)	113 (32)	1.34 (0.98–1.84)	1.23 (0.86–1.75)^a^
Leaking amniotic fluid	28 (8)	33 (9)	1.00 (0.58–1.73)	0.95 (0.53–1.70)^a^
Planned caesarean section	17 (5)	18 (5)	1.07 (0.52–2.20)	1.09 (0.49–2.40)^a^
Induction	93 (27)	86 (24)	1.13 (0.80–1.60)	1.36 (0.92–2.01)^a^
Maternal-foetal considerations**	13 (4)	43 (12)	0.25 (0.13–0.49)	0.23 (0.11–0.48)^a^
Cervical dilatation in cm on admission (labouring women), *n* = 371, mean (SD)***	4.50 (3.47)	4.11 (2.35)	1.05 (0.97–1.13)	1.02 (0.95–1.10)^a^
Induction of labour, *n* (%)	129 (37)	112 (32)	1.30 (0.95–1.79)	1.70 (1.17–2.44)^a^
Preterm birth (< 37 weeks of gestation), *n* (%)	9 (3)	25 (7)	0.38 (0.17–0.82)	0.43 (0.18–1.01)^a^
Intrapartum Oxytocin use, *n* = 645, *n* (%)****	108 (33)	101 (31)	1.08 (0.78–1.51)	0.91 (0.62–1.33)^b^
Amniotomy, *n* = 645, *n* (%)****	48 (15)	51 (16)	0.92 (0.60–1.41)	0.74 (0.44–1.23)^b^
Non-pharmacological pain-relief, *n* = 625, *n* (%)*****	239 (74)	151 (50)	2.93 (2.08–4.13)	2.95 (2.00–4.34)^c^
Pharmacological pain-relief, *n* = 625, *n* (%)*****	213 (66)	184 (61)	1.28 (0.92–1.78)	1.56 (1.02–2.38)^c^
Length of active stage of labour in hours, *n* = 549, mean (SD)******	4.93 (4.01)	4.68 (3.43)	1.02 (0.98–1.06)	1.02 (0.97–1.08)^c^

Maternal region of birth: 1) Sub-Saharan Africa, 2) Middle East, Central & South Asia, 3) Eastern Europe

Delivery site in Norway: 1) Southern Norway, 2) Western Norway, 3) Central Norway, 4) Eastern Norway

^*^Cervix ≥ 4cm and regular contractions (≥ 2/ 10 min)

^**^Reduced fetal movement, signs of pre-eclampsia or COVID-19 (including hospital referrals), persistent pain, or unplanned home birth

^***^Includes mothers in early and active labour with regular contractions (≥ 2/ 10 min)

^****^Excludes planned caesarean sections

^*****^Mothers who underwent planned caesarean sections and those who underwent acute caesarean sections without being in active labour are excluded from the data

^******^Includes only vaginal births. Active labour: cervix ≥ 4cm continuing until the birth of the baby

^a^Adjusted for: parity, age, education, pre-pregnancy BMI, maternal region of birth, and delivery site in Norway

^b^Adjusted for: a + induction of labour

^c^Adjusted for: b + oxytocin use

**Table 3:** Pain-relief during childbirth, comparing women with and without Multicultural Doula (MCD) support, crude associations, *N* = 625^a^

	**MCD support (*n* = 323)**	**No MCD support (*n* = 302)**	**OR**	**95% CI**	***P* value**
No pain-relief was administered during childbirth, *n* (%)	36 (11)	65 (22)	0.46	0.30–0.72	< 0.001
Non-pharmacological pain-relief methods, *n* (%)					
Any method	239 (75)	151 (50)	2.93	2.08–4.13	< 0.001
Warm packs	83 (26)	61 (20)	1.36	0.93–1.99	0.110
Active change of position / upright position	210 (65)	88 (29)	4.70	3.34–6.63	< 0.001
Massage	83 (26)	14 (5)	7.14	3.95–12.92	< 0.001
Shower	10 (3)	8 (3)	1.17	0.45–3.00	0.748
Bath	8 (2)	15 (5)	0.48	0.20–1.16	0.102
Transcutaneous electrical nerve stimulation (TENS)	12 (4)	9 (3)	1.25	0.52–3.01	0.620
Acupressure	5 (2)	4 (1)	1.17	0.31–4.38	0.821
Pharmacological pain-relief methods, *n* (%)					
Any method	213 (66)	184 (61)	1.28	0.92–1.78	0.151
Nitrous oxide gas	99 (31)	73 (24)	1.38	0.97–1.97	0.075
Epidural	134 (41)	127 (42)	0.97	0.70–1.33	0.837
Pudendal nerve block	47 (15)	45 (15)	0.97	0.62–1.51	0.878
Spinal	9 (3)	14 (5)	0.59	0.25–1.38	0.219
Opioids	40 (12)	17 (6)	2.36	1.31–4.27	0.004

^a^Data excludes mothers who underwent planned caesarean sections and those who underwent acute caesarean sections without being in active labour

Using the same adjustments as described above, analysing obstetric and neonatal outcomes showed that women who received MCD support had fewer emergency caesarean sections (*p* = 0.04) compared to women without MCD support (Table 4). A list of indications for emergency caesarean section is available in Supplementary File 4. Among women giving birth vaginally, those with MCD support had a significantly lower incidence of PPH than those without MCD support (*p* = < 0.001). During the immediate postpartum period, mothers who received MCD support were more likely to exclusively breastfeed at discharge (*p* = < 0.001) compared to the women without MCD support (Table 4).

**Table 4:** Associations of obstetric and neonatal outcomes, comparing women with and without Multicultural Doula (MCD) support, *N* = 678

	**MCD Support (*n* = 339)**	**No MCD support *n* = 339)**	**Univariable analysis OR or MD (95% CI)**	**Multivariable analysis Adjusted OR (95% CI)**
Mode of delivery, *n* (%)				
Normal vaginal birth	246 (73)	227 (67)	1.31 (0.94–1.81)	1.29 (0.85–1.95)^b^
Assisted vaginal delivery*	42 (12)	34 (10)	1.27 (0.79–2.05)	1.17 (0.67–2.03)^b^
Planned caesarean section	16 (5)	17 (5)	0.94 (0.47–1.89)	0.90 (0.42–1.96)^a^
Emergency caesarean section	35 (10)	61 (18)	0.53 (0.34–0.82)	0.59 (0.34–0.98)^b^
Intrauterine foetal death (IUFD), *n* (%)	3 (1)	3 (1)	1.00 (0.20–4.99)	0.82 (0.12–5.49)^b^
Perineal injury 3rd & 4th degree, *n* = 549, *n* (%)**	6 (2)	4 (3)	1.40 (0.39–5.06)	1.38 (0.34–5.54)^c^
Episiotomy, n = 549, *n* (%)**	56 (19)	55 (20)	0.89 (0.59–1.35)	0.77 (0.45–1.32)^c^
Estimated blood loss ≥ 1000ml, *n* (%)				
All births	21 (6)	56 (17)	0.34 (0.20–0.57)	0.35 (0.20–0.61)^b^
Vaginal births (*n* = 549)	12 (4)	38 (15)	0.25 (0.13–0.50)	0.25 (0.12–0.52)^b^
Caesarean section births (*n* = 129)	9 (18)	18 (23)	0.71 (0.29–1.74)	0.61 (0.19–1.92)^b^
Apgar score, mean (SD)				
5 min	9.59 (0.99)	9.45 (1.33)	1.11 (0.97–1.28)	1.11 (0.96–1.28)^d^
10 min	9.86 (0.84)	9.72 (1.08)	1.19 (0.98–1.45)	1.18 (0.97–1.43)^d^
Birth weight in grams, mean (SD)	3394 (472)	3329 (643)	1.01 (0.98–1.16)	1.00 (0.99–1.08)^a^
Breastfeeding at discharge, n = 672, *n* (%)***				
Exclusive breastfeeding	269 (80)	212 (63)	2.37 (1.67–3.35)	2.26 (1.53–3.36)^d^
Partial breastfeeding	63 (19)	107 (32)	0.49 (0.35–0.71)	0.51 (0.34–0.76)^d^
Not breastfeeding	4 (1)	17 (5)	0.23 (0.08–0.68)	0.33 (0.10–1.10)^d^

Maternal region of birth: 1) Sub-Saharan Africa, 2) Middle East, Central & South Asia, 3) Eastern Europe Delivery site in Norway: 1) Southern Norway, 2) Western Norway, 3) Central Norway, 4) Eastern Norway

^*^Includes vacuum extraction or forceps-assisted delivery

^**^Includes only vaginal births

^***^Excludes IUFD

^a^Adjusted for: parity, age, education, pre-pregnancy BMI, maternal region of birth, and delivery site in Norway

^b^Adjusted for: a + induction of labour, oxytocin use

^c^Adjusted for: b + operative/ non-operative vaginal delivery

^d^Adjusted for: b + mode of delivery

## Discussion

### Main findings

Our findings show that women receiving MCD support during pregnancy and childbirth had improved obstetric and neonatal outcomes compared to migrant women without this support, demonstrating reduced odds of acute caesarean section and PPH, greater use of pain relief during labour, and a higher rate of exclusive breastfeeding at discharge.

Strengths and limitations.

The major strength of this study is its nationwide population, covering both urban and rural areas, from all hospitals running the MCD program, spanning most of the country’s regions. This extensive coverage and relatively large study population help reduce potential selection bias, enhancing the generalisability of the findings. The study population includes > 74% refugees or asylum seekers, providing novel insights into this group, which is often excluded due to language barriers [41]. Additionally, the MCD and non-MCD groups are very similar in terms of time since immigration and other vulnerability factors, such as unemployment, low education, reasons for immigration, and interpreter needs. When present, differences favour the control group, which has a higher level of education, and higher rates of employment, European origin, and migration for family reunification, education, or work. However, many women without MCD support gave birth with only hospital personnel present, suggesting a limited social network also in this group. Standardisation of MCD training and support increases the reliability of our findings. Excluding participants without MCD presence during childbirth strengthens the validity of obstetric and neonatal findings. Conversely, limited data on the timing and quantity of MCD support during pregnancy hinders the confirmation of the trend towards reduced preterm birth, and the statistically significant differences in admission reason and induction frequency associated with MCD support.

The use of register data has limitations, relying on the accuracy of individual reporting and including a limited set of variables, potentially omitting factors that are important for a comprehensive understanding of the studied phenomenon. Securing an appropriate control group in case–control studies can be challenging. In our study, this was especially true in hospitals with efficient MCD recruitment, requiring additional effort to identify a comparison group meeting the study's inclusion criteria. A randomised controlled trial (RCT) to study the effect of MCD support was considered but ultimately deemed unsuitable. This was both due to the complexities of informing and engaging women unfamiliar with clinical trials or facing literacy challenges and ethical considerations related to withholding MCD support from migrant women in vulnerable circumstances despite the uncertain effectiveness of the intervention.

### Interpretation

Consistent with several previous studies in other contexts, we found that MCD support was associated with reduced rates of acute caesarean sections [33, 34, 42–44]. Additionally, the rate of acute caesarean sections among MCD-supported women in our study was similar to the national average in Norway in 2022 (10% vs. 9.6%)[38]. In contrast, a recent Swedish study found nulliparous doula-supported migrant women had significantly higher rates of emergency caesarean section than Swedish-born women (17.7% vs. 12.8%)[35]. The dynamics behind these differences are unknown and indicate the need for further study, such as whether MCDs have the greatest impact on birth outcomes for migrants facing significant vulnerabilities. Conflicting findings between studies also underscore the complexity of evaluating doula care across different healthcare systems and cultural settings.

Our findings align with evidence demonstrated in a Cochrane systematic review, highlighting the benefits of supplementing hospital healthcare providers with the continuity of care provided by a doula or a person chosen by the woman [30]. In contrast to usual care in Norway, where midwives attend mother-to-be in 7–8 h shifts and have their first introduction during labour, MCDs meet mothers during pregnancy and extend their support beyond midwives’ shifts during childbirth, fostering greater continuity and reducing turnover and possible stress. We posit that the presence of an MCD who shares language and cultural ties also relieves stress and creates a safe space, which has been identified as a factor in improving birth outcomes for migrants [8, 45, 46].

Our study suggests another potential mechanism contributing to the decrease in the number of caesarean sections: the role of MCDs in supporting women with prolonged deliveries. The primary cause of interventions during labour in Norway is prolonged labour, which heightens the risk of acute caesarean sections [47]. Research has underscored the importance of physical activity in childbirth progression [48]. In our study, MCD-supported mothers were significantly more active during childbirth, changing their position more frequently than the comparison group, potentially mitigating the risks associated with malposition and acute caesarean sections due to prolonged labour.

Our study found that vaginal births with MCD presence were less often complicated by PPH. While severe bleeding is a leading cause of global maternal mortality, it is largely preventable [49]; all hospitals included in the present study advocate active third-stage labour management to minimise blood loss [50]. In our study, the incidence of PPH among mothers receiving MCD support was lower than the 7.5% reported for all Norwegian births in 2020 [51], whereas the rate of significant blood loss among migrants without MCD support was considerably higher. The latter finding aligns with other research indicating increased blood loss among immigrant mothers compared to native mothers, although some studies report no significant difference [11, 13, 52]. In our study, many migrants without MCDs lacked a support person during childbirth, potentially leading to delayed assistance requests [53]. Furthermore, previous experiences of disrespectful or abusive treatment during facility births could discourage timely requests for assistance [54].

Our study found that mothers with MCD support received more pain relief compared to those without such support. Previous research has suggested that migrants are less likely to receive epidural analgesia for pain relief compared to non-migrants [14]. This difference may be influenced by various factors, including cultural beliefs and perceptions about pain management, such as whether epidural analgesia is seen as acceptable or necessary [55]. Additionally, some religious and cultural coping strategies may prioritise non-medical approaches to managing pain, which could affect a woman’s willingness or preference to use epidurals [55]. While our study found no differences between the comparison groups regarding epidural use, significant differences were observed in opioid and natural pain-relief methods use. This outcome is likely due to the MCDs encouraging mothers to adopt self-help measures, such as changing positions and requesting pain relief when needed. In a qualitative study of the Norwegian MCD program, many women reported that their doula provided emotional and physical support akin to that of a mother figure, offering massages and other forms of physical comfort as needed [28]. Ensuring good pain management is important for all women, regardless of their background, and MCD support seems to improve equality of care in this regard.

Several studies over the past ten years have shown that doula support has a positive impact on breastfeeding outcomes [32–34]. MCDs in our study proactively discussed and provided information about breastfeeding during pregnancy and post-childbirth. In addition to receiving one-day training on breastfeeding during the MCD course and their personal experiences, many doulas have pursued additional breastfeeding training. Some doulas also hold qualifications as breastfeeding counsellors. MCD support seems to positively affect mothers' motivation to breastfeed, likely by providing relevant information and lowering the threshold to seek help.

A recent mixed-method review found that community-based doula programs for migrant and refugee women in high-income countries enhance culturally responsive care, improve childbirth experiences, and potentially lead to better health outcomes [56]. Lack of cultural competence, misconceptions, and discrimination—explicit or implicit- can in turn discourage migrant women from seeking care, cause delays, resistance to interventions, misunderstandings, and lower satisfaction, while increasing stress, anxiety, and isolation [25, 26]. Addressing these issues requires a woman-centered approach, provider training, and the use of cultural mediators or multicultural doulas when a female relative or friend is unavailable, to bridge the gap between healthcare and migrant women’s cultural needs [25, 26, 57]. In this way, the use of MCDs may strengthen WHO's recommended respectful maternity care by benefiting both women and healthcare providers, fostering the provision of high-quality care [58]. However, a systematic review of community-based doulas for migrant and refugee women showed that community-based doulas best complemented the maternity care team when doula roles were clearly defined and boundaries understood by both doulas and other care providers [56].

Our study provides valuable insights into the efficacy of an MCD intervention aimed at enhancing the quality of obstetric care for migrant populations. Systematic reviews have underscored the scarcity of interventions tailored specifically to improve maternal care for migrant women, emphasising the imperative for systematic, evidence-based evaluations of such initiatives [59, 60]. Our findings suggest that MCD support can significantly enhance the quality of care for migrants, potentially allowing equitable access to healthcare despite underlying social determinants of health. These results may be useful to healthcare providers, policymakers, and stakeholders involved in funding and disseminating interventions, guiding future efforts in designing culturally competent and inclusive healthcare services for migrant populations.

## Conclusion

Our study indicates that Norway’s MCD program is associated with significantly improved obstetric and neonatal outcomes for migrant women, which positively influences women’s reproductive health and their children's well-being. This underscores the potential value of targeted interventions in improving maternal and child health within this demographic. Further studies are needed to explore the impact of MCD support on health service utilisation and the experiences and mental well-being of migrants as well as which migrant groups experience the most significant impact of MCD support on birth outcomes.

## Supplementary Information

Supplementary Material 1.

Supplementary Material 2.

Supplementary Material 3.

Supplementary Material 4.

## Data Availability

The data that support the findings of this study are not openly available due to reasons of sensitivity and are available from the corresponding author upon reasonable request. Data are located in controlled access data storage at Sørlandet Sykehus, Kristiansand, Norway. https://www.sshf.no/

## Abbreviations

Cl: Confidence interval
OR: Odds ratio
aOR: Adjusted odds ratio
MCD: Multicultural doula
PPH: Postpartum haemorrhage
