# Supplementary material for: Multicultural doula support and obstetric and neonatal outcomes: a multi-centre comparative study in Norway
Source: BMC Pregnancy Childbirth. 2024 Dec 24;24:854. doi: 10.1186/s12884-024-07073-y (PMC11667827; doi:10.1186/s12884-024-07073-y)
Supplement: Supplementary file 1 — Supplementary Material 1. [file 12884_2024_7073_MOESM1_ESM.pdf]

Supplementary file 1. Education provided to the Multicultural doulas (MCD).

Content	Amount
**Pregnancy**<br>Physiological and psychological processes of pregnancy<br>Healthcare system in Norway	1 day*
**Normal labour**<br>The physiological process of childbirth<br>Signs that labour has started<br>Transportation to the hospital<br>Interaction with healthcare professionals<br>Pharmacological and non-pharmacological pain-relief methods	1 day
**Interventions and complications during labour**<br>Induction of labour<br>Breech delivery<br>Episiotomy<br>Fetal heart rate monitoring<br>Caesarean section and vacuum extraction<br>Postpartum haemorrhage	1 day
**Breastfeeding**<br>Benefits of breastfeeding<br>Immediate skin-to-skin contact<br>Breastfeeding positions and techniques	1 day
**Post-partum period**<br>Common physical and emotional challenges<br>Postnatal depression<br>Family planning	1 day
**Other issues**<br>Circumcision<br>Violence during pregnancy<br>Different cultures, pregnancy and childbirth<br>The role of MCDs in meeting cultural, religious, or social needs<br>Ethics and confidentiality<br>Role of the Interpreter vs MCD<br>Fathers' role	1 day
**Practical training and roleplay cases**<br>The role of MCD during pregnancy, childbirth, and post-partum period<br>Relaxation- and breathing techniques<br>Massage techniques<br>Different positions during labour<br>Intrauterine fetal demise case	1 day

*1 day = 8 hours
