# Supplementary figures and images for: Multicultural doula support and obstetric and neonatal outcomes: a multi-centre comparative study in Norway
Source: BMC Pregnancy Childbirth. 2024 Dec 24;24:854. doi: 10.1186/s12884-024-07073-y (PMC11667827; doi:10.1186/s12884-024-07073-y)

Supplementary file 2.

A flowchart showing the reasons leading to exclusion from the study.

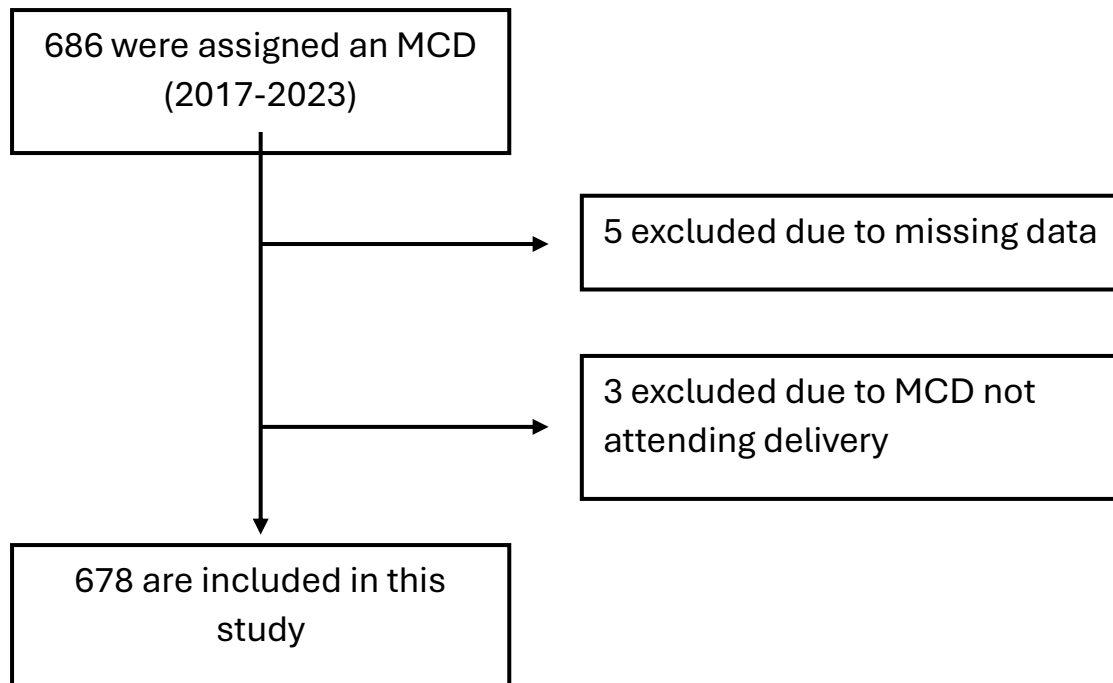

Supplement: Supplementary file 2 — Supplementary Material 2. [file 12884_2024_7073_MOESM2_ESM.pdf]
