# Supplementary material for: Multicultural doula support and obstetric and neonatal outcomes: a multi-centre comparative study in Norway
Source: BMC Pregnancy Childbirth. 2024 Dec 24;24:854. doi: 10.1186/s12884-024-07073-y (PMC11667827; doi:10.1186/s12884-024-07073-y)
Supplement: Supplementary file 3 — Supplementary Material 3. [file 12884_2024_7073_MOESM3_ESM.pdf]

Supplementary file 3.

Participants came from 52 countries and spoke 36 languages:

Arabic (29%), Tigrinya (18%), Dari (8%), Somali (8%), Ukrainian (5%), Amharic (4%), Urdu (3%), Polish (2,2%), Swahili (2%), Turkish (1,3%), and less than 1% spoke each Farsi, Romanian, Bengali, Persian, Hindi, Tamil, Kurdish, Pashto, Thai, French, English, Albanian, Bulgarian, Greek, Chinese, Croatian, Kurmanji, Lithuanian, Malaysian, Nepalese, Oromo, Punjabi, Portuguese, Russian, Spanish or Vietnamese.
