# Supplementary material for: Multicultural doula support and obstetric and neonatal outcomes: a multi-centre comparative study in Norway
Source: BMC Pregnancy Childbirth. 2024 Dec 24;24:854. doi: 10.1186/s12884-024-07073-y (PMC11667827; doi:10.1186/s12884-024-07073-y)
Supplement: Supplementary file 4 — Supplementary Material 4. [file 12884_2024_7073_MOESM4_ESM.pdf]

Supplementary file 4.

Reasons for emergency caesarean sections comparing women with an without Multicultural Doula (MCD) support

Reasons for an emergency caesarean section	MCD support	No MCD support
Failure to progress	15	22
Pre-eclampsia/eclampsia/HELLP syndrome	2	1
Admitted before planned caesarean section due to uterine constraction.	1	1
Fetal concerns (pathological CTG)	6	18
Umbilical cord prolapse		4
Placenta previa	1	2
Suspected or confirmed uterine rupture	1	3
Placental abruption		4
Circumcised, previously repaired, now in active labor	1	
Failed vacuum extraction	4	1
Maternal infection	3	1
History of previous traumatic birth experience		1
Missing	1	1
